# Stomach Pain Upon Stomach Pain: Medication-Induced Pancreatitis

**DOI:** 10.7759/cureus.35554

**Published:** 2023-02-27

**Authors:** Ethan K Chambers, Eugeniu Stratulat, Gurjeet Judge, Seaf Shafique, Luisa Ladel

**Affiliations:** 1 Internal Medicine, Norwalk Hospital, Norwalk, USA

**Keywords:** medication side-effects, drug-related side effects and adverse reactions, mercaptopurine, pancreatitis and ibd, medication-induced pancreatitis

## Abstract

As a first-line immunosuppressant to maintain remission in Crohn's disease, 6-mercaptopurine (6-MP) has been commonly used. A rare, unpredictable, dose-independent and idiosyncratic reaction to this medication is acute pancreatitis. Unlike other side effects of this drug which have been well characterized and are often dose-dependent, acute pancreatitis is an uncommon adverse effect not frequently encountered in clinical practice.

In this case report, we describe a 40-year-old man with Crohn's disease who developed acute pancreatitis within two weeks of starting 6-MP. Discontinuation of the drug followed by fluid resuscitation led to the overall improvement of symptoms within 72 hours. No complications were noted during the follow-up. It is our intention to raise awareness for this lesser-known side effect with this case report and to urge physicians to provide thorough counseling prior to starting on this medication, especially in patients with inflammatory bowel disease (IBD).

Additionally, we hope to reinforce this disease entity as a differential for acute pancreatitis and aim to emphasize the importance of detailed medication reconciliations with this report, especially in the emergency department, to enable quick diagnoses and limit unnecessary treatments.

## Introduction

Azathioprine (AZA), 6-mercaptopurine (6-MP), and 6-thioguanine represent a group of purine-based antimetabolite drugs called thiopurines. Metabolism of AZA after ingestion leads to the production of 6-MP, thereby making it a prodrug [1]. AZA and 6-MP are widely known for their use in the treatment of inflammatory bowel disease (IBD) and are commonly used for the maintenance of remission in patients with ulcerative colitis (UC) or Crohn’s disease [2]. Their mechanism of action is based on their ability to inhibit cellular proliferation, in this case targeting lymphocytes by limiting their differentiation and thus reducing inflammatory response [3].

As with any drug therapy, adverse reactions must be anticipated and mitigated if present. Some of the dose-dependent adverse reactions include myelosuppression and hepatic toxicity, which are often manageable with dose reduction [4]. Common idiosyncratic or non-dose-dependent reactions include intractable nausea, fever without leukopenia, and arthralgia [5]. These adverse reactions often do not respond to dose reduction and frequently warrant termination of therapy [6]. Another much less common idiosyncratic adverse drug reaction of thiopurines is acute pancreatitis. Approximately 3% of patients with IBD develop pancreatitis within the first month after initiation of therapy with 6-MP and AZA [2]. Interestingly, IBD patients appear to be more likely to develop this side effect than other patient groups on thiopurines [7].

The exact mechanism of 6-MP and AZA-induced pancreatitis remains unknown, few mechanisms have been postulated including its effect on the thiopurine methyltransferase activity. An immune-mediated genetic predisposition and delayed type 2 versus type 4 allergic reaction have also been proposed. Recent data supporting the latter ties in with evidence of pancreatitis recurrence after the re-introduction of AZA [5]. In this study, we report a case of 6-MP-induced acute pancreatitis in an otherwise healthy patient with Crohn’s disease.

## Case presentation

A 40-year-old male with a past medical history significant for Crohn's disease and cholecystectomy secondary to a common bile duct stone presented to the emergency room with acute-onset abdominal pain associated with nausea and vomiting. On admission, the patient was afebrile and hemodynamically stable. He described an eight-hour history of left upper quadrant discomfort, not associated with any food intake, which then wandered and progressed to high-intensity epigastric pain radiating to his back. He also reported one episode of non-bloody, bilious emesis, and persistent nausea. His admission labs were significant for leukocytosis of 12x109/L with neutrophilic predominance, normocytic anemia of 12.8 g/dL, mildly elevated liver panel with a cholestatic pattern, namely alkaline phosphatase of 162 U/L, gamma-glutamyl transferase (GGT) of 94 U/L, total bilirubin of 0.8 mg/dL, alanine transaminase (ALT) of 45 U/L, aspartate transaminase (AST) of 58 U/L, and a significantly elevated lipase at 1,708 U/L. Triglycerides were normal at 86 mg/dL. Electrolytes and kidney function were within normal limits as well. C-reactive protein was elevated at 130.4 mg/L.

**Figure 1:**
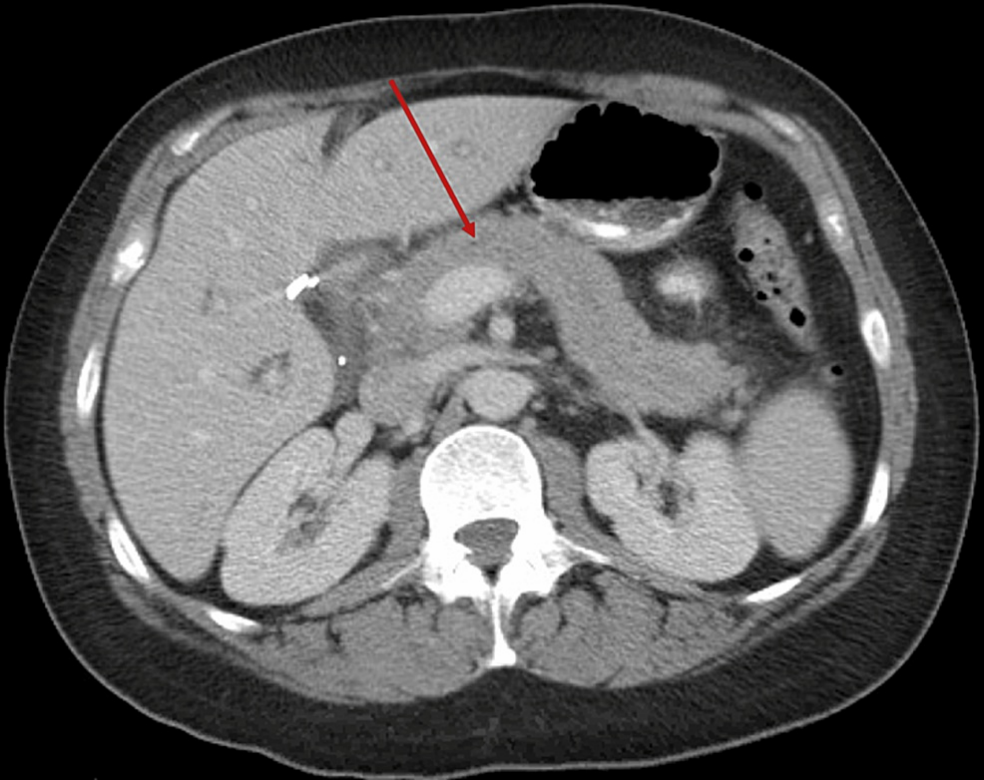
Computed tomography (CT) imaging of abdomen/pelvis, transverse section, showing inflammation of the pancreas and surrounding area consistent with pancreatitis (red arrow).

Given the patient’s characteristic symptoms and elevated lipase level greater than three times the upper limit of normal, a diagnosis of mild acute pancreatitis was made. Computed tomography (CT) imaging (Figure 1) of the abdomen and pelvis showed mild peripancreatic and periportal edema as well as peripancreatic fat stranding without evidence of pseudocysts, abscesses, or necrosis. Given the absence of any signs of organ failure, this was classified as mild acute pancreatitis.

The patient was placed on high-volume intravenous fluids, intravenous pain management and a clear liquid diet and was admitted to the medical service. Further questioning revealed that the patient underwent a cholecystectomy approx. four years prior, which greatly reduces the likelihood of a gallstone as the cause of this pancreatitis, consistent with a lack of bilirubinemia on admission. The patient’s lipid panel including triglycerides was within normal limits, and while he did have an endoscopic retrograde cholangiopancreatography (ERCP) about four years prior, post-ERCP pancreatitis would be very unlikely given the long interval. Therefore, both hypertriglyceridemia-induced and post-procedure pancreatitis were ruled out. In addition, the patient reported seldom intake of alcohol, with his last drink about a month prior to admission. In line, ALT and AST did not appear elevated and ethanol level on admission was undetectable, making alcohol-induced pancreatitis highly unlikely. However, the patient had recently been started on 6-mercaptopurine about two weeks prior to admission for treatment of Crohn’s disease. Given the low probability of other causes as detailed above, a diagnosis of drug-induced pancreatitis was made.

6-mercaptopurine, which the patient was taking at a dose of 50mg once daily, was held upon admission. The patient did not have any symptoms concerning an acute flare of Crohn’s so there was no need to emergently start other immunosuppressive therapy. In terms of his pancreatitis, he recovered with supportive treatment of fluid resuscitation and pain management. Within 24 hours there was no further need for opioid analgesia and the patient was advanced to a low-fat diet within 48 hours which he tolerated well. The patient was able to be discharged within 72 hours of admission. During follow-up with the patient’s gastroenterologist after discharge 6-mercaptopurine was discontinued indefinitely and the patient was started on Infliximab for treatment of his Crohn’s disease.

## Discussion

Our patient's presentation is in many ways a classic example of 6-mercaptopurine (6-MP) induced acute pancreatitis. Firstly, our case presents a patient with Crohn's disease and thiopurine-induced pancreatitis (TIP) cases are known to occur more commonly in Crohn’s disease than in other inflammatory conditions [8]. Furthermore, our patient presented within three weeks of the commencement of the treatment, which is in line with previous reports, and had been started on a dose of 50mg daily which is the same dosage noted to have caused TIP in previous reports [9]. Our patient also presented with overt signs of pancreatitis, including increased pancreas enzyme laboratory values, epigastric pain, and evidence of pancreatitis on imaging, and the patient's pancreatic enzymes and epigastric pain quickly improved after the offending drug was discontinued. Interestingly, our patient differed from previously described presentations in his gender, as most case reports evidence the development of TIP in females and children [8]. 

TIP is a rare, however well-known adverse effect of thiopurine medications when used for the treatment of autoimmune diseases. It is notable that patients with Crohn’s disease appear especially sensitive to this particular drug reaction. Not only do patients with Crohn's disease have a 4.3 times higher risk of developing acute pancreatitis when compared to healthy volunteers [7]. Teich et al. found that patients suffering from Crohn’s disease also had a 2.7-fold increased risk of TIP when compared to patients with ulcerative colitis, the most common IBD entity [6]. Gordon et al. placed the number to treat (NNT) to encounter one case of acute 6-MP-induced pancreatitis between 31 and 36 depending on treatment indication and patient group [10].

Reasons as to why the incidence of 6-MP-induced pancreatitis is so much higher in patients with Crohn’s disease are currently unclear but may be found in its pathophysiology. The Crohn’s disease profile, unlike other IBD, includes duodenal manifestations and pancreatic manifestations, and the increased prevalence of additional autoimmune disorders. Interestingly, Teich et al. found an increased risk of TIP in active smokers and smoking is a known risk factor for developing Crohn’s disease as well. Furthermore, it is known to increase the risk of complications during Crohn’s disease, such as a higher frequency of strictures, fistulas, and increased need for surgery [6].

As for pathophysiology, Gordon et al. showed that there is a dose-independent relationship between thiopurine application and the development of pancreatitis. Other side effects of thiopurine usage, such as myelosuppression, occur in a dose-dependent fashion. This dose-independent relationship seen in TIP suggests an etiology independent of thiopurine methyltransferase activity [10]. Haber et al. postulated that this finding may be explained by an allergic-type mechanism in thiopurine-induced pancreatitis. This hypothesis is supported by the frequent occurrence of disease three weeks after the beginning of therapy, and almost immediate recurrence after re-exposure. These findings together with a lack of allergic skin reactions or eosinophilia are suggestive of type III or type IV hypersensitivity mechanism [11].

Overall, pancreatitis caused by a medication-related adverse effect is rare and only responsible for <5% of all acute pancreatitis episodes. However, many drugs have been reported to be able to trigger drug-induced pancreatitis, although not all work through the same mechanisms. Some, such as 6-MP, sulfonamides, and aminosalicylates cause inflammation through immunologic reactions. Valproic acid, Pentamidine, and Tetracycline on the other hand cause pancreatitis via the accumulation of toxic metabolites. Other medications have a direct toxic effect and/or cause ischemia to the pancreas including diuretics, sulfonamides, and azathioprine [11], and some are known to cause either intravascular thrombosis (estrogens) or increased viscosity of pancreatic secretions, both leading to stasis and blockade in the pancreatic duct with subsequent inflammatory reaction. 6-MP is categorized as a Class Ib drug, signifying that there are multiple case reports of pancreatitis with positive rechallenge, although other causes, such as alcohol, hypertriglyceridemia, gallstones, and other drugs could not be ruled out [12].

## Conclusions

Additional research is required to better understand the pathophysiology of 6-MP-induced acute pancreatitis and possibly develop tools of prediction for which patients might be more likely to develop drug-induced pancreatitis beyond generic risk factors, such as pre-existing conditions and overall comorbidity burden. As in our case, even young patients with little to no significant comorbidities are at risk and predisposing factors remain unclear. Prescribers should be distinctly aware of this potentially life-threatening side effect, especially in patients with Crohn’s disease, and should counsel patients accordingly prior to treatment start. Finally, this report highlights how accurate medication reconciliations can aid in quick diagnostics and help to avoid unnecessary treatment applications for the patient and our healthcare system.
